# Novel Bidirectional ESD Circuit for GaN HEMT

**DOI:** 10.3390/mi16020129

**Published:** 2025-01-23

**Authors:** Pengfei Zhang, Cheng Yang, Jingyu Shen, Xiaorong Luo, Gaoqiang Deng, Shuxiang Sun, Yuxi Wei, Jie Wei

**Affiliations:** 1 School of Integrated Circuit Science and Engineering, University of Electronic Science and Technology of China, Chengdu 610054, China; pengfei.zhang@std.uestc.edu.cn (P.Z.); chengyang_0510@foxmail.com (C.Y.); sunshuxiang@huanghuai.edu.cn (S.S.); yuxi.wei@foxmail.com (Y.W.); weijieuestc@uestc.edu.cn (J.W.); 2 China Resources Microelectronics (Chongqing) Limited, Chongqing 401331, China; shenjy513@foxmail.com; 3 College of Microelectronics, Chengdu University of Information Technology, Chengdu 610225, China

**Keywords:** ESD, GaN, diode, p-GaN HEMT

## Abstract

In this paper, the ESD protection circuit for p-GaN gate HEMTs with bidirectional clamp is proposed and investigated. ESD clamp circuits consist of several forward diodes in serials and a reverse diode. During the ESD pulse, a discharging channel in the proposed ESD clamp is built and the gate to source voltage for p-GaN HEMTs is clamped at safety value. Based on the experimental verification, the proposed ESD clamps have bidirectional protection functionality by being triggered by a required voltage and exhibit a high secondary breakdown current in both forward and reverse transient ESD events. Meanwhile, the proposed ESD clamp circuit can decrease the power loss in a static state.

## 1. Introduction

With superior working properties, the p-GaN gate high-electron mobility transistor (HEMT) has emerged as a prominent power device market. Meanwhile, its gate structure can be easily damaged by electrostatic discharge (ESD) events [1,2,3,4]. Recently, some articles have reported the ESD robustness of GaN HEMTs [5,6,7,8]. The commercial p-GaN gate HEMTs need to reach an industrial standard of more than 2 kV human body model (HBM) failure voltage (*V*_HBM_), which is equal to human body 1.5 kΩ, multiplied by secondary breakdown current (*I*_S_) (≥1.34 A) [9]. Therefore, incorporating ESD protection devices or circuits into GaN power systems has become a practical choice. Nevertheless, the p-GaN gate HEMTs equivalent *V*_HBM_ for the gate-to-source condition is only 0.2∼0.33 kV [10]. Consequently, it is necessary to improve its gate-to-source ESD robustness by ESD circuit. Furthermore, since the protected HEMT also faces the threat of reverse electrostatic discharging current [11], reverse discharging properties should be taken into consideration during ESD protection circuit design.

However, there are only a few reports on ESD-protected circuit design for GaN HEMTs. An integrated GaN-based ESD protection structure is proposed by diodes in serials [12]. Nevertheless, the circuit limits to a unidirectional protection functionality and occupies a substantial chip area. Zener diode [13] has bidirectional clamp capability, but it is still incompatible with existing p-GaN HEMT technology. The resistive ESD protection circuit has bidirectional protection functionality [14], but the resistance value becomes important, referring to the trade-off between chip area and leakage current. The ESD additional power dissipation significantly impacts the overall performance and energy efficiency of the system, which is a crucial design concern. In addition, the combination of diodes with resistors can achieve high performance [15,16]. They also face a similar dilemma as resistive ESD protection circuits. As for capacitor ESD protection circuits [17,18,19], on the one hand, those circuits have relatively low leakage current, and, on the other hand, those circuits involving stray parameters during the transient situation lead to a small design margin. ESD circuits in recent fully integrated GaN designs [20,21] have more than one discharging device. Those clamps increase discharging resistance and require high device consistency.

In this work, we proposed novel self-triggered ESD discharging circuits, which consist of diodes, a current-limiting resistor and a p-GaN HEMT. The proposed ESD clamps can protect bidirectional ESD conditions and satisfy the industrial standard of bidirectional ESD robustness requirements. This work is organized as follows. Firstly, the structures and mechanisms of two ESD circuits are presented. Subsequently, the characteristics of the proposed ESD clamps are investigated. Finally, the impact of the proposed ESD circuit on the switching characteristics of p-GaN HEMTs is given.

## 2. Structure and Mechanism

**Figure 1:**
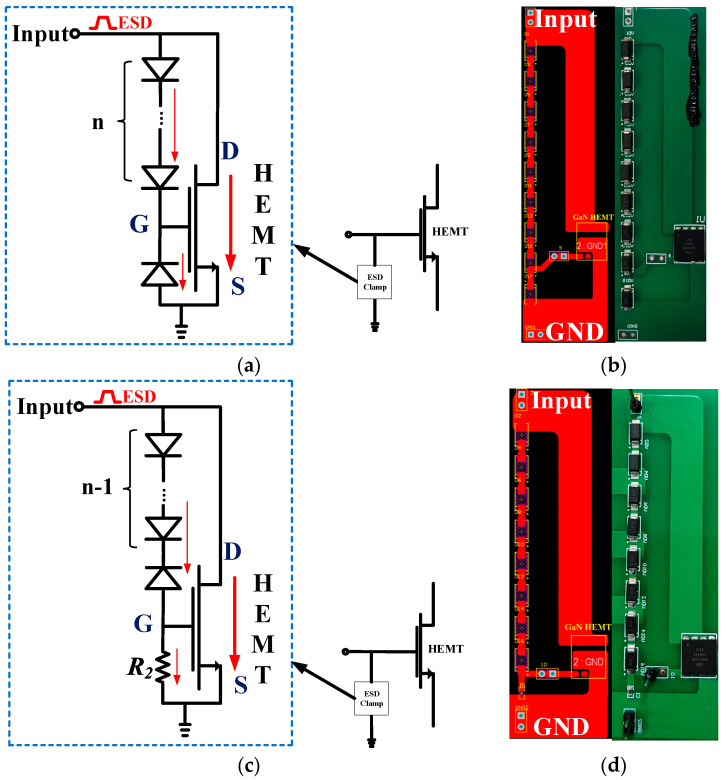
Clamp1: (**a**) schematic structure, (**b**) PCB layout and picture; Clamp2: (**c**) schematic structure, (**d**) PCB layout and picture.

Two bidirectional ESD circuits are proposed in this work. Compared to the resistive ESD clamp circuit, the low leakage current can be realized by incorporating a reverse diode into ESD clamps. Figure 1a gives the schematic ESD configuration (designated as clamp1). It consists of several diodes and a p-GaN HEMT. Multiple forward diodes are connected in series between the gate and drain of the HEMT, while a reverse diode is connected between the gate and source. Figure 1c,d show the second proposed topology (designated as clamp2). All the devices in the proposed ESD circuits could be readily integrated into p-GaN technology, making ESD design more convenient. Meanwhile, the gate-source shorted p-GaN HEMT may also be used as the diodes of clamp2. Given that conducting the experiment is more economical, equivalent circuit topology is printed on a circuit board (PCB) to verify the feasibility of circuit topology. Figure 1b,d are, respectively, clamp1’s and clamp2’s PCB layout and PCB picture. In clamp2, several forward diodes with one reverse diode are connected in series between the gate and drain and a current-limiting resistor is parallelly connected between the HEMT’s gate and the source.

When the Input node in Figure 1 meets a forward pulse of an ESD event under dynamic conditions, the forward current flows through the forward diodes, reverse diode or R_2_. Then, a transient voltage is built at the G node. Since the Input node obtains an increasing current from the ESD pulse, the voltage of the G node rises over the threshold voltage (*V*th) of HEMT in the ESD clamp. It triggers the HEMT to turn on. Consequently, the forward electrostatic discharging current can flow through the ESD circuit, effectively clamping the gate-to-source voltage of the main protected HEMT within a safe operational range. The main protected HEMT is mainly at a steady state during its operating lifetime, and thus, the static condition of ESD protection circuits should also be taken into consideration.

The forward diode has an equivalent resistance (*r*_on_), and the reverse diode shows dynamic equivalent resistance (*r*_off_). The reverse diode’s static equivalent resistance approaches infinity (∞) as its parasitic capacitor has been fully charged. Therefore, Equations (1) and (2) show the proportional relationship between *V*_Tri_ and *V*th of clamp1. As shown in Equations (3) and (4), the *V*_Tri_ is different between transient state and static state for clamp2. Generally, the forward triggering voltage should be larger than the gate operating voltage of 5 V of the protected HEMT in order not to disturb normal operation [22]. For p-GaN gate HEMT, its gate bias allowed for long-term reliable operation is approximately 8V [23]. The optimal *V*_Tri_ is between 5 V and 8 V. Equation (2) indicates that clamp1 clamps the gate of protected HEMT around *V*th under static conditions regardless of the number of diodes, which cannot realize ESD protection. Nevertheless, Equations (3) and (4) theoretically verified the feasibility of clamp2.

clamp1(1)Transient:VTri≈ Vth×(1+nronroff)(2)Static:VTri≈ Vth×∞+nron∞≈ Vth

clamp2(3)Transient:VTri ≈ Vth×1+roff+n−1ronR2(4)Static:VTri≈ Vth×∞+n−1ron+R2R2≈ ∞

To experimentally validate the proposed design at a low cost, an equivalent structure has been constructed on PCB. The diodes (RS1A) with *r*_on_ around 44 mΩ and the commercial p-GaN HEMTs (INN700D240B) with *V*th around 1.7 V are used in PCB test experiments. The transient ESD events are simulated by the transmission line pulsing (TLP) measurement system HED-T5000 (HANWA, Tokyo, Japan) and the TLP pulses are configured to have a duration of 100 ns and a rise time of 10 ns time referring to previous ESD studies [24]. Furthermore, the bidirectional TLP current–voltage (*I*–*V*) characteristics of the proposed ESD clamps are extracted. Meanwhile, its bidirectional static *I*–*V* characteristics are also extracted.

## 3. Results and Discussion

**Figure 2:**
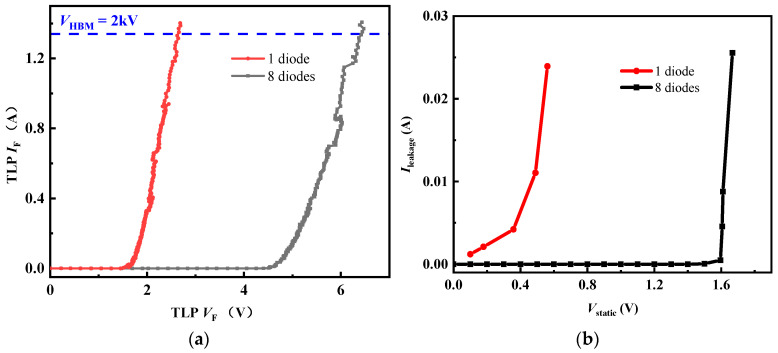
(**a**) Positive TLP *I–V* characteristics of clamp1; (**b**) positive leakage current characteristics of clamp1.

Figure 2a shows the TLP *I*–*V* curves of clamp1 with a different number of forward diodes. During the ESD charges zapping at the Input node, clamp1 with 8 and 1 forward diodes could be triggered at 4.7 V and 1.8 V, respectively. It verifies that *V*_Tri_ positively correlated to the number of diodes. In addition, the two circuits both possessed a high secondary breakdown current of more than 1.34 A. It demonstrates that clamp1 could effectively release the accumulated electrostatic charges with the appropriate number of forward diodes. Nevertheless, the static *I–V* curves in Figure 2b show unacceptable low static *V*_Tri_ (lower than the gate operating voltage 5 V), and thus clamp1 cannot protect the main HEMT. By the way, static *V*_Tri_ decreases with the number of forward diodes. This could be explained by that the input static signal of the test machine may not filter out all the alternating signals.

**Figure 3:**
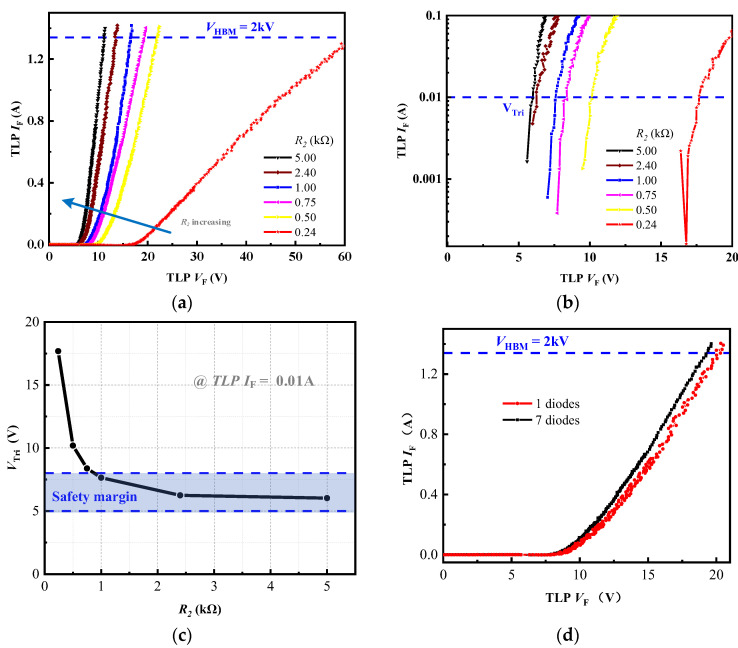
Test result for clamp2: positive TLP *I–V* characteristics with different *R*_2_ values (**a**) linear-scale and (**b**) log-scale; (**c**) *V*_Tri_; (**d**) positive TLP *I–V* characteristics with different number of forward diodes.

Figure 3a shows that clamp2 can achieve more than 2 kV HBM failure voltage in a positive TLP test. With the increasing *R*_2_, *V*_Tri_ decreases from 17.7 V to 6 V and its decrease trend is decelerating in Figure 3b,c. Moreover, the curves in Figure 3a have an increasing slope when *R*_2_ rises, indicating an enhanced discharging efficiency. Given the ESD clamp design safety margin, a desirable *R*_2_ value should be chosen from 1 kΩ to 5 kΩ under the condition of 5 V ≤ V_Tri_ ≤ 8 V. As illustrated in Figure 3d, the number of forward diodes has little impact on the discharge characteristics because *r*_on_ (~mΩ) is several orders of magnitude smaller than *R*_2_ (~kΩ).

**Figure 4:**
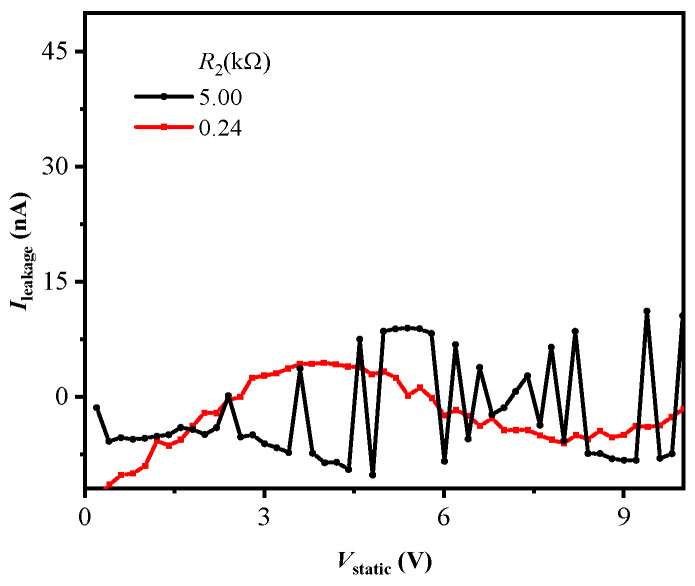
Positive leakage current characteristics of clamp2 with different *R*_2_ values.

Figure 4 gives the static leakage current of clamp2 with different values of *R*_2_. Clamp2 keeps leakage current at nano-ampere (nA) order, which proves static equivalent resistance of the reverse diode is also far larger than *r*_off_, as shown in Equation (4). Meanwhile, compared to resistive ESD clamp with milliampere (mA) leakage current [14], clamp2 decreases additional power dissipation.

**Figure 5:**
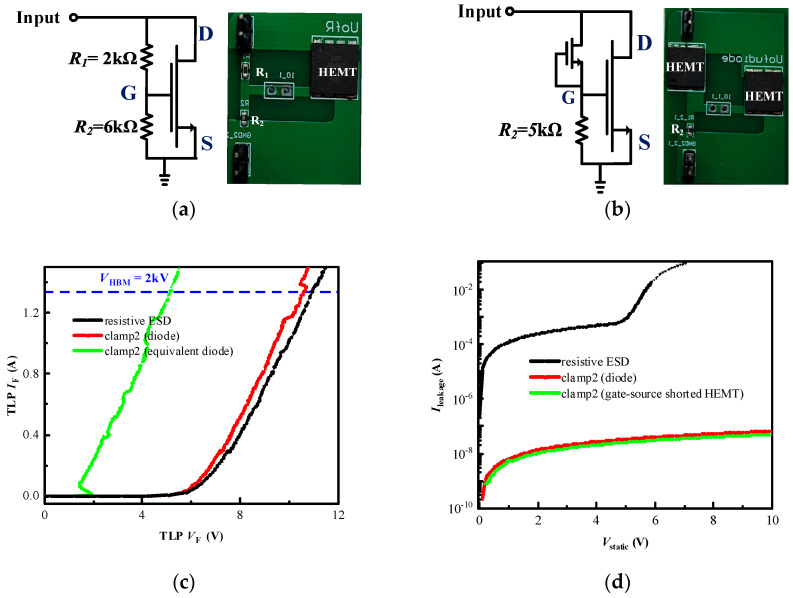
Schematic structure and test board pictures of (**a**) resistive ESD circuit and (**b**) clamp2 with gate-source shorted p-GaN HEMT as equivalent diode; comparison of test results of: (**c**) positive TLP *I–V* and (**d**) positive leakage current characteristics with different ESD circuit.

Figure 5 compares the positive TLP *I–V* and positive leakage current characteristics with a resistive ESD circuit, clamp2 with RS1A and clamp2 with gate-source shorted p-GaN HEMT. (Since forward diodes of clamp2 have little impact on the circuit characteristics, the forward diodes or equivalent diodes of clamp2 are removed.) The clamp2 with RS1A and clamp2 with gate-source shorted p-GaN HEMT have the same R_2_ (5 kΩ) at the bias circuit. The conventional resistive ESD circuit has R_1_ (2 kΩ) and R_2_ (6 kΩ) at the bias circuit. All three ESD circuits show more than 1.34 A discharging property under the TLP test. Nevertheless, when it comes to static leakage property, two types of clamp2 reduce by more than five orders of magnitude. It proves that clamp2 design for ESD circuits can significantly reduce additional power dissipation.

We also investigate reverse TLP *I–V* characteristics of clamp2. Its reverse TLP *I–V* characteristics depend on *R*_2_. Its reverse *V*_Tri_ is evaluated in Equation (5).

(5) $$\mathrm{Reverse}\;\mathrm{TLP}\text{:}\;V_{\mathrm{Tri}}\;\approx \;V_{\mathrm{th}}\times \left(1+\frac{R_{2}}{r_{\mathrm{off}}+\left(n-1\right)r_{\mathrm{on}}}\right)$$

**Figure 6:**
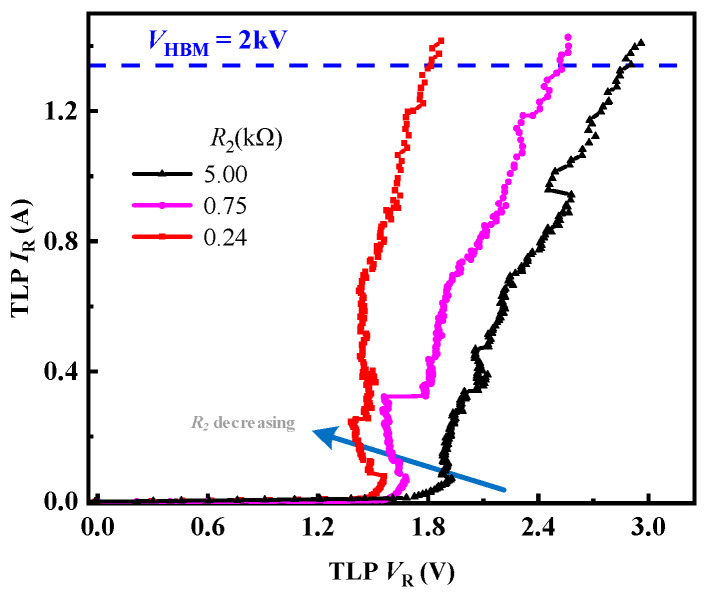
Reverse TLP *I–V* characteristics of clamp2.

Decreasing *R*_2_ leads to diminishing *V*_Tri_, which is opposite to forward TLP *I–V* characteristics. Since the protected HEMT does not operate on reverse bias conditions, the reverse safety margin of the ESD clamp is 0 V to 8 V. Figure 6 shows that the clamp2 could be triggered by low voltage and achieve an *I*_S_ more than 1.34 A under reverse TLP condition. It reveals that *R*_2_ is higher than *r*_off_. Meanwhile, reverse *V*_Tri_ fully locates at the safety margin when *R*_2_ changes from 0.24 kΩ to 5.0 kΩ. A desirable *R*_2_ value should be chosen from 1 kΩ to 5 kΩ, taking the forward and reverse ESD protection into consideration.

**Figure 7:**
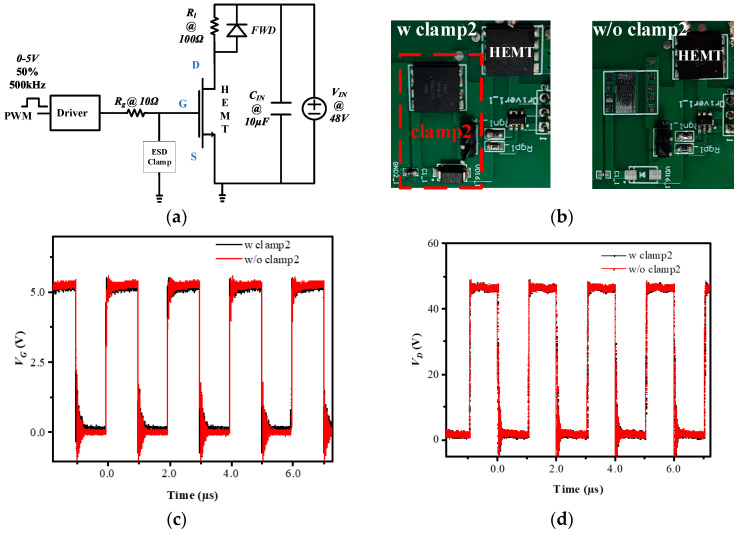
Switch characteristics of protected HEMT with or without the proposed ESD clamp2: (**a**) schematic structure; (**b**) test board pictures; (**c**) test result for gate voltage of mainHEMT (*V*_G_); (**d**) test result for drain voltage of mainHEMT (*V*_D_).

To verify the affection of clamp2 to the switching characteristic of the high-current and high-power p-GaN HEMTs, clamp2 is put into a test board circuit as shown in Figure 7. Figure 7a is the schematic structure of the whole circuit, where *R_g_* is the resistor between the driver and the main HEMT gate electrode, *R_l_* is the load resistor, the *FWD* is the commercial flywheel diode, *C_IN_* is the filter capacitor and *V_IN_* is the supply voltage in the test circuit. The test PWM (pulse-width modulation) pulse has a duty circled about 50%, and its switching frequency is 500 kHz. Figure 7c,d show that the switching waveforms about the gate voltage of mainHEMT (*V*_G_) and the drain voltage of mainHEMT (*V*_D_) of two test boards coincide at the same switching condition. It indicates that the proposed clamp2 has a negligible impact on the switching characteristic in its application.

## 4. Conclusions

The novel ESD protection circuits are proposed to enhance the ESD robustness of a GaN power system in both forward and reverse directions. Through the TLP tests, it is demonstrated that ESD protection circuits possess more than 2 kV of V_HBM_ in transient ESD events. Clamp2 not only has a triggered voltage in safety margin (5~8 V), but also has superior power loss property. We found that the required triggering voltages of clamp2 are strongly related to *R*_2_. As *R*_2_ decreased from 5 kΩ to 1 kΩ, the forward triggering voltages increased from 6 V to 8 V, and the reverse triggering voltages decreased from 1.9 V to 1.6 V. In addition, the proposed ESD clamps can be easily integrated with p-GaN HEMT, demonstrating a good reference for the ESD design fully compatible with the GaN monolithic fabricating process.

## Data Availability

The original contributions presented in this study are included in the article. Further inquiries can be directed to the corresponding authors.
